# Effect of Explant Source on Phenotypic Changes of In Vitro Grown Cannabis Plantlets over Multiple Subcultures

**DOI:** 10.3390/biology12030443

**Published:** 2023-03-13

**Authors:** Mohsen Hesami, Kristian Adamek, Marco Pepe, Andrew Maxwell Phineas Jones

**Affiliations:** Department of Plant Agriculture, University of Guelph, Guelph, ON N1G 2W1, Canada

**Keywords:** biotechnology, culture decline, leaf morphology, marijuana, multiplication, plant tissue culture, prolonged culture, rejuvenation, somaclonal variation

## Abstract

**Simple Summary:**

While micropropagation systems have been developed for cannabis, little attention has been given to the potential impact of explant source within the plant on subsequent growth and development in vitro. From other species, it is known that the explant source can have a significant impact on future development, presumably due to variations in endogenous phytohormones, sugars, and epigenetic state. This study was performed using a high-cannabinoid cultivar of cannabis, an economically important crop, to compare the growth and development of explants collected from various positions within the plant, ranging from the top to the bottom. Our results show that the multiplication rate (i.e., number of nodes), especially with explants derived from middle and apical parts of the mother plant, decreased over multiple subcultures, which can result from epigenetic machinery. In contrast, explants derived from the basal portion of the plant had an acceptable multiplication rate throughout the experiment and produced cannabis plantlets with shorter but wider leaves, indicative of rejuvenation. Hence, these results suggest that basal-derived cannabis explants should be used preferentially as starting material for in vitro propagation.

**Abstract:**

Drug-type cannabis is often multiplied using micropropagation methods to produce genetically uniform and disease/insect-free crops. However, micropropagated plantlets often exhibit phenotypic variation, leading to culture decline over time. In cannabis, the source of these changes remains unknown, though several factors (e.g., explant’s sources and prolonged in vitro culture) can result in such phenotypical variations. The study presented herein evaluates the effects of explant sources (i.e., nodal segments derived from the basal, near-basal, middle, and apical parts of the greenhouse-grown mother plant) over multiple subcultures (4 subcultures during 235 days) on multiplication parameters and leaf morphological traits of in vitro cannabis plantlets. While initial in vitro responses were similar among explants sourced from different regions of the plant, there were significant differences in performance over the course of multiple subcultures. Specifically, explant source and/or the number of subcultures significantly impacted plantlet height, number of nodes, and canopy surface area. The explants derived from the basal and near-basal parts of the plant resulted in the tallest shoots with the greatest number of nodes, while the explants derived from the middle and apical regions led to shorter shoots with fewer nodes. Moreover, the basal-derived explants produced cannabis plantlets with shorter but wider leaves which demonstrated the potential of such explants for in vitro rejuvenation practices with minimal culture decline. This study provides new evidence into the long-term impacts of explant source in cannabis micropropagation.

## 1. Introduction

Cannabis (*Cannabis sativa* L.) is a dioecious, herbaceous crop with a variable growth habit [1] that is widely cultivated for its medicinal [2], nutritional [3], industrial [4], and ornamental [5] potentials. Cannabis is conventionally propagated through either sexual (i.e., seed) or asexual (e.g., cuttings) methods [1]. There are issues associated with both methods. Cannabis propagation through seeds is typically challenging due to high levels of heterozygosity [6]. Conventional cutting methods used for cannabis are time-consuming and associated with a wide range of diseases (e.g., viruses, bacteria, and fungi) that hamper the phytosanitary quality of the plants obtained [7]. While vegetative propagation through cuttings theoretically produces plants that are phenotypically and genetically identical to their mother plants, cannabis producers have noted that cuttings taken from mother plants change over time and generally become less vigorous with lower levels of cannabinoids [8].

Micropropagation is an application of plant tissue culture that represents an alternative and promising approach for large-scale cannabis propagation of genetically uniform plants with high phytosanitary quality that can be produced in confined areas [6,7,9]. In addition to mass propagation, plant tissue culture is fundamental to biotechnology, allowing researchers to study developmental biology [10,11], secondary metabolite production [12], bioenergy production [13], cryopreservation [14], and transgenic- and genome-editing-based methods [15]. However, culture decline is a problem commonly encountered in tissue culture, which refers to the gradual loss of vigour and capacity for growth in the cultured cells and/or tissues [16]. Reasons for culture decline are multifactorial and can include genetic factors, epigenetic changes, physiological changes, microbial contamination, and environmental stressors [17,18].

Cannabis has presented challenges in micropropagation including high rates of hyperhydricity, low multiplication rates, and culture decline over time [19]. These challenges are highly genotype specific, and new approaches are needed to develop methods that work across diverse genetic backgrounds. While most research has focused on optimizing culture conditions to overcome these challenges, little attention has been given to the source of starting material and if this has an impact on subsequent growth and development in culture. From other species, we know that long-term explant performance can be impacted by various factors related to the explant such as season [20], developmental stage [21], and location within the plant [22].

These differences are presumably due to variations in endogenous plant growth regulators, sugars/resources, and epigenetic regulation [23]. Therefore, the explant source plays a fundamental role in culture decline over time. Generally, juvenile explants have several benefits in growth vitality and are generally more responsive to micropropagation techniques in comparison with mature explants [24,25]. For example, despite being chronologically older, nodal segments closer to the base of a plant are typically more juvenile than nodal segments on the apical part, and explants from the basal part generally have better responses to in vitro rejuvenation practices [26]. Indeed, based on the explant source, in vitro plant rejuvenation is accompanied by specific changes in gene expression patterns and epigenetic regulation, which finally lead to different phenotypical changes such as phyllotaxy, the shape and size of leaves, as well as the capacity for multiplication [26]. In relation to cannabis, Adamek et al. [8] previously showed that the accumulation of somatic mutations in top-part-derived cuttings was significantly higher than in bottom-part-derived cuttings from the cannabis mother plant, showing the importance of explant source. The physical position and age of the explant largely impact the composition of internal phytohormones, which can influence tissue responsiveness [27]. Additionally, tissue size, type, and developmental phase represent explant characteristics that affect competence for response induction and overall success [28]. Hence, the explant source can be considered one of the most important factors in rejuvenation practices to overcome culture decline over time.

Apart from the source of the explants, the number of subcultures also plays a pivotal role in culture decline [29,30]. The integrity of the developing in vitro plantlet can often only be evaluated after several rounds of subculture [31] and a comprehensive recognition of morphogenic tissue responses is often required to assess in vitro efficiency [32]. Though the impacts of explant material and culture period on culture decline are clear, there is no study investigating the role of explant sources and number of subcultures on culture decline in cannabis during in vitro multiplication.

Detection of the best source of explants and the number of subcultures to overcome culture decline over time plays a fundamental role in the characterization of in vitro multiplicated cannabis plantlets for future use in commercial cultivation. Therefore, the current study evaluates the effect of explant sources and the number of subcultures on morphological parameters of in vitro multiplicated cannabis plantlets.

## 2. Materials and Methods

### 2.1. Explant’s Source Description

**Figure 1:**
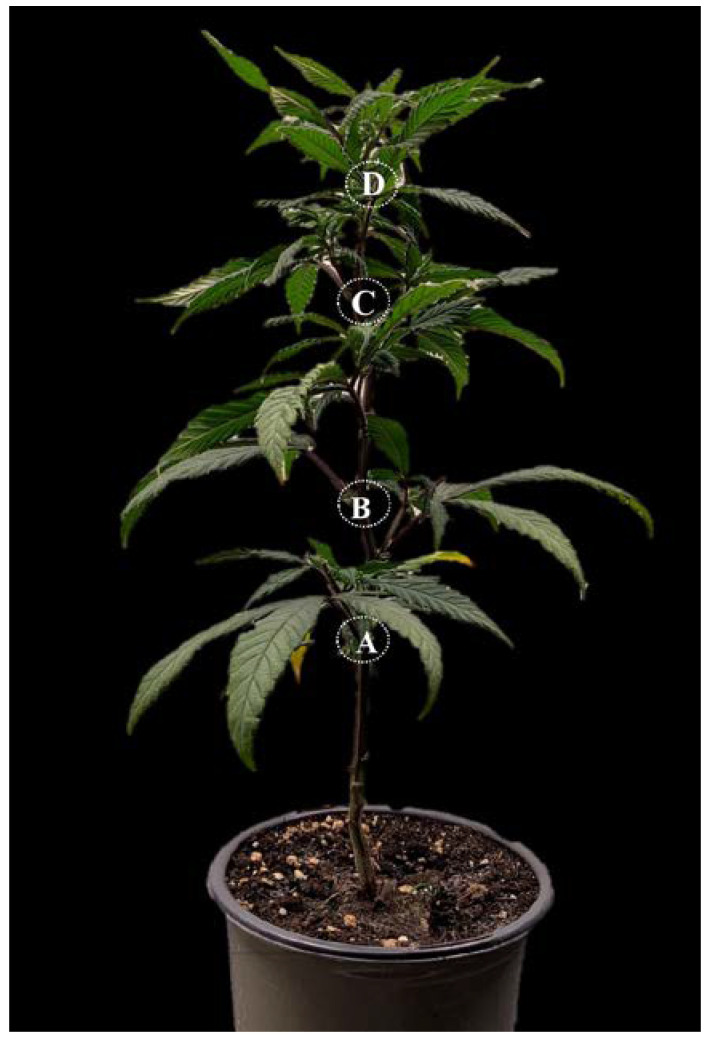
Four different sources of the explants include the basal part (A), near-basal part (B), middle part (C), and apical part (D) of the greenhouse-grown mother plant.

Stem segments with two buds derived from a drug-type of cannabis (Honey Banana) were selected as explants, using the greenhouse-grown mother plant. Since Honey Banana responds poorly to in vitro culture compared with other cannabis cultivars, it can be considered a potential candidate to study the effect of the explant’s sources on in vitro performance and rejuvenation during prolonged in vitro culture. To study the effect of the explant’s sources, stem segments with two nodes were selected from different parts of the greenhouse-grown mother plant (Figure 1) including the basal part (A), near-basal part (B), middle part (C), and apical part (D).

The plant used in this study was cut from a maintained mother plant aged slightly over 2.5 years and was provided with the ideal environment to enable the mother plant to thrive. The mother plant originally came from seed but was renewed from a clone at about the 2-year mark to reinvigorate the plant. The mother plant was transferred to appropriately sized pots as it grew until it was maintained in a 76 L (20 gallon) pot with nutrient-rich soil. A nutrient solution designed for vegetative growth was supplied every 3–5 d to maintain appropriate moisture levels in the soil with an adjusted pH of ~6.5. It contained high levels of nitrogen, with moderate levels of phosphorous, potassium (N-P-K), and micronutrients. Environment conditions consisted of temperatures between 20–25 °C with relative humidity of 55–65%. The mother plant was kept under long-day photoperiods of 18:6 hr light/dark cycles to sustain an indefinite vegetative state. The light source was 300 W broad white-spectrum lights (4.2% red 650–670 nm) with a photosynthesis photon flux of >440 μmol s^−1^ and a PAR photon efficacy of 2.2 μmol J^−1^.

### 2.2. In Vitro Culture Condition and Origin of Subcultures

Driver and Kuniyaki Walnut (DKW) culture medium [33] (D2470, PhytoTech Labs, Lenexa, KS, USA) supplemented with 30 g/L sucrose and 6 g/L agar (Thermo-Fisher Scientific, Waltham, MA, USA) was used as a basal medium in this study. The pH of the medium was adjusted to 5.8 before autoclaving for 20 min at 121 °C.

The decontamination of the explants was performed using 10% commercial bleach with 1.4 mL/L Tween 20 (*v*/*v*) (7 drops per 500 mL of solution) for 10 min followed by 3 rinses with sterile distilled water (each time 5 min). Each sterilized explant was cultured in a Magenta GA7 vessel (Fisher Scientific, NJ, USA) containing 40 mL of basal DKW medium and kept in the growth chamber at 25  ±  2 °C under a 16 h photoperiod with 40  ±  5 μmol m^−2^ s^−1^ light intensity.

To study the effect of the number of subcultures, 4 consecutive subcultures were conducted including subculture I after 45 days, subculture II after 60 days, subculture III after 60 days, and subculture IV after 70 days. Before each subculture, the plantlets were defoliated and stem segments with two nodes were used as an explant. After each subculture, several traits (i.e., plantlet height, number of nodes, number of shoots, and canopy surface area) were measured.

Several morphological traits of the leaf (i.e., number of leaflets, number of primary serrations of the central leaflet, length of the central leaflet, width of the central leaflet, and length/width ratio of the central leaflet) were measured using five samples (leaves) from each treatment.

### 2.3. Experimental Design and Statistical Analysis

The experiment was performed based on a completely randomized design with 2 replicates in a 4 × 4 factorial design (4 sources of the explants and 4 subcultures). The data were analyzed by analysis of variance (ANOVA) followed by Duncan’s multiple range test at *p* < 0.05. SAS version 9.3 was also used for data analysis. The morphological traits of the leaf were analyzed using ImageJ.

## 3. Results

**Figure 2:**
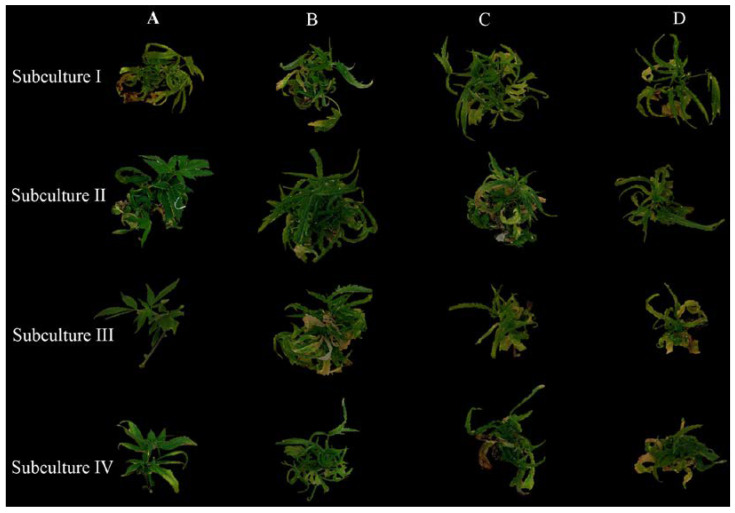
Effect of the source of explants and number of subcultures on in vitro multiplication of cannabis. The explants derived from the basal, near-basal, middle, and apical parts are respectively illustrated as A, B, C, and D.

In the current investigation, the impacts of the source of explants and the number of subcultures on in vitro multiplication of cannabis were assessed (Figure 2). Based on our results (Table 1), plantlet height, number of nodes, and canopy surface area were significantly influenced by either the source of explants or the number of subcultures. However, the interaction of the source of explants and the number of subcultures had no significant effect on plantlet height and the number of nodes. In addition, neither a single factor nor interaction significantly affected the number of shoots (Table 1). All the explants exhibited hperhydricity after 45 days in culture (subculture I). Additionally, all the explants were hyperhydric throughout subculture II (60 days following subculture I), with the exception of explants derived from the basal part (Figure 2). Throughout subcultures III and IV (60 days and 130 days following subculture II, respectively), only explants derived from the middle and apical parts exhibited hyperhydricity (Figure 2).

**Figure 3:**
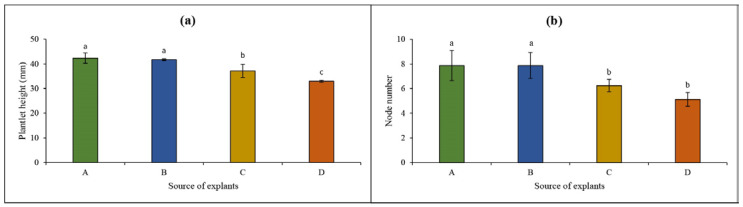
Single effect of the source of explants on (**a**) plantlet height and (**b**) number of nodes during in vitro multiplication of cannabis. Each value (column) shows the average of the explant source effect in all subcultures. Error bars represent standard error.

Our results also showed that the maximum plantlet height was obtained from the explants derived from the basal part (42.34 mm) and the near-basal part (41.72 mm), which was significantly greater than the middle (37.13 mm) and apical (32.97 mm) parts (Figure 3a). Similar results were observed for the number of nodes where 7.87, 7.87, 6.25, and 5.12 nodes were respectively obtained from the basal, near-basal, middle, and apical parts (Figure 3b). Generally, these results illustrated that explants taken from basal and near-basal parts of the mother plant can result in better in vitro morphological performance in comparison with other parts.

**Figure 4:**
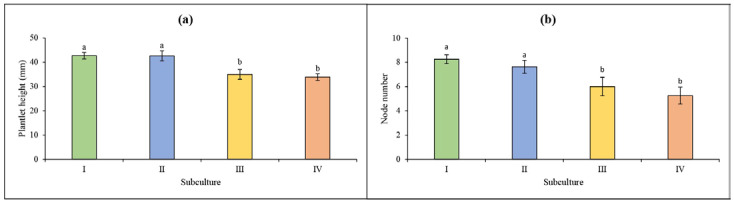
Single effect of the number of subcultures on (**a**) plantlet height and (**b**) number of nodes during in vitro multiplication of cannabis. Each value (column) shows the average of the subculture effect in all explants. Error bars represent standard error.

Our results (Figure 4a) also showed that the highest plantlet height was observed in subcultures I (42.73 mm) and II (42.61 mm), which was significantly higher than subcultures III (34.98 mm) and IV (33.86 mm). Similar results were observed for the number of nodes where 8.25, 7.62, 6.00, and 5.25 nodes were respectively observed in subcultures I, II, III, and IV (Figure 4b).

**Figure 5:**
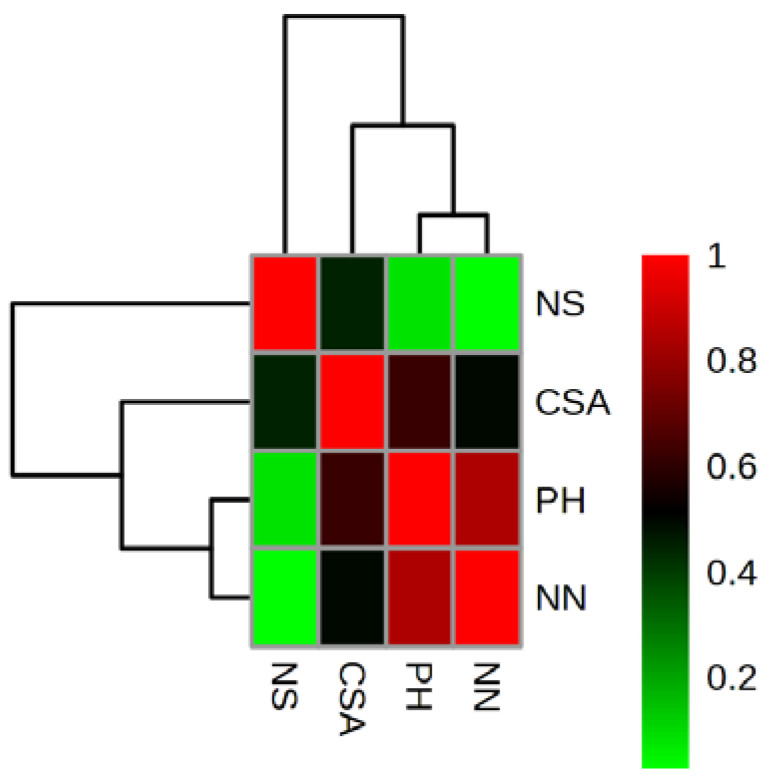
Heat map correlation among the in vitro morphological parameters (plant height (PH), number of nodes (NN), number of shoots (NS), and canopy surface area (CSA)) during in vitro prolonged culture of cannabis explants derived from different parts of the greenhouse-grown mother plant.

According to the correlation coefficient results (Figure 5), a significant positive correlation was obtained from the canopy surface area with other morphological parameters (i.e., number of nodes, number of shoots, and plant height). In addition, plant height had a significant positive correlation with the number of nodes and canopy surface area (Figure 5). The number of nodes also significantly positively correlated with the plant height and canopy surface area (Figure 5). However, the number of shoots had only a significant positive correlation with canopy surface area (Figure 5). Generally, it can be concluded that the multiplicated plantlets with higher node numbers as well as shoot number and length lead to greater canopy surface area.

**Figure 6:**
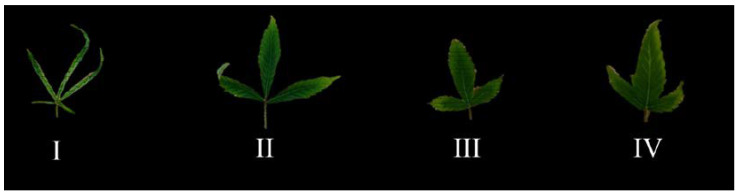
Effect of the number of subcultures (i.e., I, II, III, and IV) on leaf anatomy of in vitro grown cannabis.

In addition, our results demonstrated that the plantlet’s leaves from all the explant’s sources in subculture I consisted of five leaflets (Table 2). However, the number of leaflets decreased to 3 leaflets in later subcultures (Figure 6). The results also showed that the length of the central leaflet decreased during prolonged in vitro culture in all explants (Table 2). However, the number of primary serrations for all explants had fluctuated trends in each subculture (Table 2). Although the width of the central leaflet increased during prolonged in vitro culture, this trait fluctuated during subcultures II and III (Table 2).

**Figure 7:**
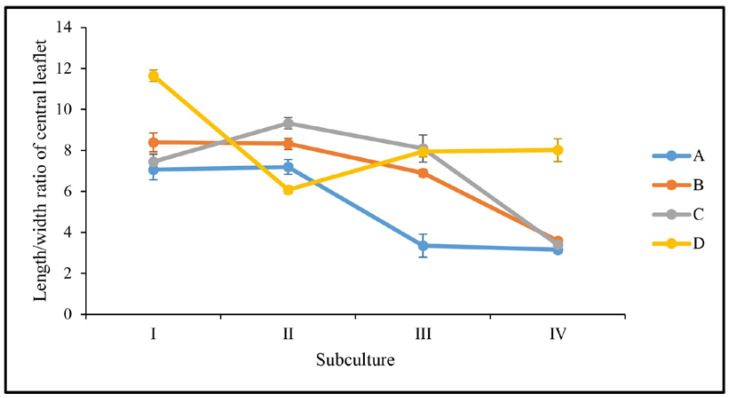
Effect of the number of subcultures (i.e., I, II, III, and IV) and the source of explants on length/width ratio of the central leaflet. The explants derived from the basal, near-basal, middle, and apical parts are respectively been illustrated as A, B, C, and D. Error bars represent standard error.

Prolonged in vitro culture by several subcultures resulted in in vitro grown plantlets with shorter but wider leaves, indicative of rejuvenation (Figure 7). Although the length/width ratio of the central leaflet decreased during prolonged in vitro culture, each explant had different trends (Figure 7). For instance, the ratio in the explants derived from the basal part was significantly decreased from subculture II to subculture III (Figure 7). However, the ratio in the explants derived from apical parts increased from subculture II to subculture IV, which might be due to the hyperhydricity (Figure 7). Our results showed that the explants derived from the basal part are more amenable to rejuvenation.

## 4. Discussion

Appropriate explant selection is the key initial stage of micropropagation [7]. Although there is no comprehensive study on the effect of explant source on cannabis multiplication, studies in other plant species demonstrate the fundamental importance that the source of the explant plays in successful in vitro multiplication and overcoming culture decline [23]. Hence, in the current study, different sources of nodal segments were selected from the basal, near-basal, middle, and apical parts of the greenhouse-grown mother plant. Based on our results, the explants derived from the basal and near-basal parts led to maximum plantlet height, number of nodes, and canopy surface area. Our results also display that the explants derived from the basal and near-basal parts produced plantlets of higher quality, which shows that these types of explants have greater potential to produce more vigorous plantlets. Similar to our results, Mestinšek-Mubi et al. [34] showed that basal-derived nodal explants resulted in maximum shoot length and node numbers, which were significantly greater than apical-derived explants in two drug-type cannabis genotypes (MX-CBD-11 and MX-CBD-707). Comparable results were also reported with other plant species such as *Salvia tomentosa* [21], *Rauvolfia serpentina* [35], *Quercus robur* [36], *Persea americana* [37], *Solanum tuberosum* [38], *Ginkgo biloba* [20], *Rubus fruticosus* [39], *Caralluma lasiantha* [40], *Psidium guajava* [41], *Hemidesmus indicus* [42], *Ficus religiosa* [43], and *Cedrela montana* [44] which showed that explants derived from basal parts had better performance than those apical-derived explants. The endogenous balance of phytohormones in the explants obtained from the basal and near-basal parts of the mother plant contributes to the emergence of their juvenile characteristics [35,45]. Therefore, such explants have been reported as the best source of initial explants for in vitro rejuvenation practices [21,38,40,44]. Moreover, such explants have better performance in tackling the adverse effects of somaclonal variations [23,46,47]. Alternatively, somaclonal variation can sometimes serve as a mechanism for genetic improvement [48]. Thus, choosing the appropriate explant should be dependent on the application of interest and, perhaps, while basal and near-basal-derived cannabis explants show value for multiplication, middle and apical explants would be more suitable for genetic improvement applications.

Apart from the source of the explants, the number and duration of subcultures play a fundamental role in the successful in vitro regeneration [49,50]. Various methods (e.g., morphological, physiological, molecular, and biochemical markers) can be used for detecting somaclonal variations [29,30,47]. Our results showed that the number of nodes and plantlet height significantly decreased after four subcultures. In particular, the number of nodes in the explants derived from middle and apical parts is approximately halved in subculture IV compared with subculture I. In line with our results, Khan et al. [51] showed a decrease in the multiplication rate and an increase in somaclonal variation frequency of *Musa sapientum* after eight subcultures. Similar results were also reported in *Olea europaea* [52] and *Coffea arabica* [53]. Previous studies showed that prolonged in vitro culture-induced DNA hypermethylation ultimately changes the expression of morphogenic genes which can result in a decline in multiplication rate [54,55,56,57]. Therefore, a reduction in the number of nodes of cannabis plantlets after several subcultures can be considered a morphological sign of somaclonal variation, which, in our case, is explicitly shown by explants derived from the middle and apical parts of the cannabis mother plant.

Since explants are typically selected from mature greenhouse-grown mother plants, the explants undergo a rejuvenation process during tissue culture practices [26]. It has also been well documented that changes in morphological traits of the leaf (e.g., number of leaflets, length of the central leaflet, and width of the central leaflet) correlate with developmental phases (i.e., vegetative, transition, and reproductive) in cannabis plants [58,59,60,61]. Therefore, leaf morphological changes can be used as a sign of in vitro rejuvenation [61]. In the vegetative phase of cannabis development, the number of leaflets typically increases from one leaflet in the seedling stage to roughly nine in the mature stage [58]. Our results showed that the number of leaflets in all explants decreased from five to three leaflets after the second subculture, suggesting rejuvenation. Our results also showed that prolonged in vitro culture resulted in cannabis plantlets with shorter but wider leaves, another sign of rejuvenation. However, explants derived from the apical part produced plantlets with longer but shorter leaves after the second subculture, which demonstrates that plantlets derived from the apical part are less amenable to the rejuvenation process. It has been reported that nodal segments closer to the base of a plant are typically more juvenile than nodal segments on the apical part, and explants from the basal part generally have better responses to in vitro rejuvenation practices [26]. Bastías et al. [62] investigated the role of explant source on in vitro plant rejuvenation in *Prunus* sp. plants by studying miRNA172 and miRNA156/157 expression patterns and several of their target genes during rejuvenation. Higher expression of miR156 and miR157 was observed in the leaves of juvenile explants than in mature explants, while the opposite was observed for the expression of miR172 [62]. These findings showed that explants originating from the juvenile part can overcome culture decline over time [62]. While the source of this phenomenon is not known, several studies in other plant species reported this as the result of epigenetic changes associated with rejuvenation [23,26,46,47,63]. For instance, Fraga et al. [24] reported significant differences in the levels of DNA methylation between meristematic regions of mature and juvenile leaves of *Pinus radiata* plants. The level of DNA methylation in meristematic areas of mature leaves was higher than in juvenile leaves. The modification in the level of DNA methylation during rejuvenation showed that plant rejuvenation can be considered a result of epigenetic changes which are dependent on the explant source [24]. Our results also showed that the length/width ratio of the central leaflet significantly decreased, especially in plantlets derived from basal and near-basal parts. Therefore, it can be concluded that explants derived from basal and near-basal parts of the mother plant are considerably more appropriate initial explants for in vitro rejuvenation and multiplication. Similar to our results, previous studies using other plant species showed that the source of the explants plays a pivotal role in successful in vitro rejuvenation [26,64,65,66,67,68,69,70].

## 5. Conclusions

This study aimed to evaluate the explant source and number of subcultures on in vitro rejuvenation and phenotypic variations of cannabis plantlets. Our results showed that the number of nodes, especially in the explants derived from middle and apical parts, decreased during prolonged in vitro culture which can be a result of epigenetic somaclonal variation. Moreover, leaf morphology analysis showed that the explants derived from the basal part of the mother plant were better suited to be initial explants for in vitro cannabis rejuvenation practices. Since previous studies on other plant species, such as potatoes [38], showed that the effects of explant source on in vitro multiplication and somaclonal variation are genotype dependent, further investigations should be evaluated using different cannabis cultivars. Although this study demonstrates the importance of explant source on initial and long-term health of cannabis cultures, further studies with greater replications are needed. Future studies are also needed to assess molecular aspects of somaclonal variation in cannabis plantlets derived from different explant sources throughout prolonged periods of in vitro culture.

## Figures and Tables

**Table 1:** Effect of the source of explants and number of subcultures on plantlets height (PH), number of nodes (NN), number of shoots (NS), and canopy surface area (CSA) during in vitro multiplication of cannabis.

Subculture	Explant’s Source	PH ± SE (mm)	NN ± SE	NS ± SE	CSA ± SE (mm^2^)
I	A	43.45 ± 1.113	8.5 ± 0.501	1 ± 0.000	994 ± 31.093
I	B	45.335 ± 2.192	8.5 ± 0.501	1 ± 0.000	1004 ± 5.015
I	C	43.88 ± 2.648	8.5 ± 1.504	1 ± 0.000	1296 ± 60.179
I	D	38.25 ± 2.848	7.5 ± 0.501	1 ± 0.000	1119.5 ± 45.636
II	A	49.71 ± 1.525	8.5 ± 0.501	1 ± 0.000	1927.3 ± 74.221
II	B	43.995 ± 2.533	8.5 ± 1.504	1.5 ± 0.501	2293.5 ± 57.672
II	C	41.21 ± 2.046	7.5 ± 0.501	1 ± 0.000	1392 ± 67.200
II	D	35.475 ± 2.071	6 ± 1.003	1 ± 0.000	1025 ± 129.385
III	A	39.33 ± 0.923	7.5 ± 0.501	1 ± 0.000	1124.5 ± 58.675
III	B	41.005 ± 1.439	8 ± 1.003	1.5 ± 0.501	1388 ± 19.057
III	C	30.36 ± 1.033	5 ± 1.003	1 ± 0.000	889.5 ± 15.546
III	D	29.22 ± 0.802	3.5 ± 0.501	1 ± 0.000	803 ± 22.066
IV	A	36.865 ± 2.553	7 ± 1.003	1 ± 0.000	1182.5 ± 46.639
IV	B	36.54 ± 1.113	6.5 ± 1.504	1 ± 0.000	968.5 ± 37.612
IV	C	33.075 ± 1.449	4 ± 1.003	1 ± 0.000	957 ± 21.063
IV	D	28.995 ± 0.707	3.5 ± 0.501	1 ± 0.000	967.5 ± 46.639
*p* value					
Explant		<0.0001	0.0010	0.5847	<0.0001
Subculture		<0.0001	0.0014	0.1546	<0.0001
Explant × subculture		0.1400	0.6394	0.7271	<0.0001

A: Explant derived from basal part; B: explant derived from near-basal part; C: explant derived from middle part; D: explant derived from apical part; SE: standard error.

**Table 2:** Effect of the source of explants and number of subcultures on phenotypical parameters of leaf.

Subculture	Explant’s Source	NLl ± SE	LCLl ± SE (mm)	WCLl ± SE (mm)	NPS ± SE
I	A	5 ± 0.000	44.20 ± 1.249	6.36 ± 0.375	18.4 ± 0.400
I	B	5 ± 0.000	47.30 ± 0.870	5.70 ± 0.329	21.6 ± 0.748
I	C	5 ± 0.000	49.53 ± 2.249	6.67 ± 0.260	22.8 ± 1.020
I	D	5 ± 0.000	58.81 ± 0.695	5.06 ± 0.151	19.2 ± 0.800
II	A	3 ± 0.000	40.66 ± 1.373	5.68 ± 0.212	20.8 ± 0.490
II	B	3 ± 0.000	40.76 ± 2.141	4.89 ± 0.178	21.2 ± 0.800
II	C	3 ± 0.000	42.19 ± 0.527	4.54 ± 0.149	17.2 ± 0.490
II	D	3 ± 0.000	45.94 ± 1.172	7.58 ± 0.296	19.2 ± 0.490
III	A	3 ± 0.000	33.37 ± 1.741	9.99 ± 0.297	20.8 ± 0.490
III	B	3 ± 0.000	34.60 ± 1.001	5.11 ± 0.330	16.4 ± 0.400
III	C	3 ± 0.000	40.85 ± 0.763	5.05 ± 0.097	20.8 ± 0.490
III	D	3 ± 0.000	42.27 ± 0.660	5.43 ± 0.328	18.8 ± 0.490
IV	A	3 ± 0.000	27.07 ± 1.339	8.57 ± 0.179	18.4 ± 0.400
IV	B	3 ± 0.000	35.30 ± 0.664	9.85 ± 0.303	18 ± 0.000
IV	C	3 ± 0.000	39.25 ± 1.242	11.60 ± 0.604	18 ± 0.000
IV	D	3 ± 0.000	40.44 ± 0.787	5.12 ± 0.283	19.2 ± 0.490

A: Explant derived from basal part; B: explant derived from near-basal part; C: explant derived from middle part; D: explant derived from apical part; LCLl: length of central leaflet; NLl: number of leaflets per leaf; NPS: number of primary serrations on central leaflet; SE: standard error; WCLl: width of central leaflet.

## Data Availability

All relevant data are within the paper.
